# Characterization of ACE Inhibitors and AT_1_R Antagonists with Regard to Their Effect on ACE2 Expression and Infection with SARS-CoV-2 Using a Caco-2 Cell Model

**DOI:** 10.3390/life11080810

**Published:** 2021-08-10

**Authors:** Philipp Reus, Ann-Kathrin Schneider, Thomas Ulshöfer, Marina Henke, Denisa Bojkova, Jindrich Cinatl, Sandra Ciesek, Gerd Geisslinger, Volker Laux, Mira Grättinger, Philip Gribbon, Susanne Schiffmann

**Affiliations:** 1 Fraunhofer Institute for Translational Medicine and Pharmacology (ITMP), Theodor-Stern-Kai 7, 60596 Frankfurt am Main, Germany; philipp.reus@itmp.fraunhofer.de (P.R.); ann-kathrin.schneider@itmp.fraunhofer.de (A.-K.S.); thomas.ulshoefer@itmp.fraunhofer.de (T.U.); marina.henke@itmp.fraunhofer.de (M.H.); sandra.ciesek@kgu.de (S.C.); geisslinger@em.uni-frankfurt.de (G.G.); volker.laux@itmp.fraunhofer.de (V.L.); Mira.Graettinger@itmp.fraunhofer.de (M.G.); Philip.Gribbon@itmp.fraunhofer.de (P.G.); 2 Institute of Medical Virology, University Hospital Frankfurt, Goethe University, Paul-Ehrlich-Str. 40, 60596 Frankfurt am Main, Germany; Denisa.Bojkova@kgu.de (D.B.); cinatl@em.uni-frankfurt.de (J.C.); 3 Pharmazentrum Frankfurt/ZAFES, Department of Clinical Pharmacology, Goethe-University Hospital Frankfurt, Theodor-Stern-Kai 7, 60590 Frankfurt am Main, Germany; 4 Fraunhofer Cluster of Excellence Immune Mediated Diseases, CIMD, 60596 Frankfurt am Main, Germany

**Keywords:** ACE inhibitor, AT1 receptor antagonist, SARS-CoV-2, cell barrier integrity

## Abstract

Blood-pressure-lowering drugs are proposed to foster SARS-CoV-2 infection by pharmacological upregulation of angiotensin-converting enzyme 2 (ACE2), the binding partner of the virus spike (S) protein, located on the surface of the host cells. Conversely, it is postulated that angiotensin–renin system antagonists may prevent lung damage caused by SARS-CoV-2 infection, by reducing angiotensin II levels, which can induce permeability of lung endothelial barrier via its interaction with the AT_1_ receptor (AT_1_R). Methods: We have investigated the influence of the ACE inhibitors (lisinopril, captopril) and the AT_1_ antagonists (telmisartan, olmesartan) on the level of ACE2 mRNA and protein expression as well as their influence on the cytopathic effect of SARS-CoV-2 and on the cell barrier integrity in a Caco-2 cell model. Results: The drugs revealed no effect on ACE2 mRNA and protein expression. ACE inhibitors and AT_1_R antagonist olmesartan did not influence the infection rate of SARS-CoV-2 and were unable to prevent the SARS-CoV-2-induced cell barrier disturbance. A concentration of 25 µg/mL telmisartan significantly reduced the virus replication rate. Conclusion: ACE inhibitors and AT_1_R antagonist showed neither beneficial nor detrimental effects on SARS-CoV-2-infection and cell barrier integrity in vitro at pharmacologically relevant concentrations.

## 1. Introduction

The coronavirus disease 2019 (COVID-19) caused by the severe acute respiratory syndrome coronavirus (SARS-CoV-2) infection raises several challenges for the treatment of patients, especially for individuals with underlying illnesses such as hypertension. There are contradictory hypotheses on the potential benefit–risk profiles of antihypertensive drugs [1,2] that act on the renin–angiotensin system for the treatment of COVID-19 patients. It is estimated that there are 1.13 billion people with high blood pressure worldwide [3] and half of COVID-19 patients develop hypotension during their hospitalization [4,5], so it is essential to clarify whether this drug group has beneficial or detrimental effects on SARS-CoV-2 infection.

The angiotensin II receptor (AT)_1_ antagonists losartan, telmisartan, and olmesartan are widely used for the control of hypertension and kidney disorders and are known as relatively safe drugs [6,7]. The angiotensin-converting enzyme (ACE) blockers ramipril, captopril, and lisinopril, also known for their relatively low side effects, are used worldwide for the treatment of chronic illnesses such as hypertension and diabetes [8].

ACE converts angiotensin I into angiotensin II, which mediates vasoconstriction and blood pressure-increasing effects via the AT_1_-receptor. Therefore, AT_1_R antagonists and ACE inhibitors are frequently used for the control of hypertension. ACE2, in turn, is an aminopeptidase membrane protein, which converts angiotensin II into angiotensin 1–7 and intervenes in a regulating manner in the cardiovascular system by reducing the angiotensin II concentration. ACE2 is highly expressed on the surface of heart and lung cells and is used by coronaviruses such as SARS-CoV and SARS-CoV-2 to enter host cells [9].

In this context, it is currently discussed whether ACE inhibitors or AT_1_R antagonists facilitate the entry of SARS-CoV-2 into host cells, as these drugs may possibly lead to an upregulation of ACE2. On the other hand, SARS-CoV-2 reduces the expression of ACE2 and thereby leads to an increase in angiotensin II. Angiotensin II, in turn, can increase permeability in alveoli epithelial cells via binding to the AT_1_R and could thereby promote the damage of lung tissue [10]. Antagonizing of AT_1_R could prevent SARS-CoV-2-increased permeability. It is therefore important to evaluate the effects of these classes of drugs (ACE inhibitors, AT_1_R antagonists) on the progression and outcome of SARS-CoV-2 infection.

The aim of this study was to investigate whether antagonists of AT_1_R such as telmisartan and olmesartan as well as ACE inhibitors such as captopril and lisinopril could influence SARS-CoV-2 infection and/or the permeability of cell barriers. The Caco-2 model was used as a test system, since these cells express ACE2 and can be readily infected by SARS-CoV-2 [11]. We investigated the effect of antagonists of AT_1_ and ACE inhibitors on the viral replication, ACE2 mRNA, and protein expression levels, cell barrier integrity and the viability of Caco-2 cells.

## 2. Materials and Methods

### 2.1. Cells and Reagents

Caco-2 cells were purchased from Sigma Aldrich (Schnelldorf, Germany) and cultured in minimum essential Eagle medium supplemented with 10% FCS (heat-inactivated at 56 °C), 2 mM L-glutamine and 1x non-essential amino acid solution. All media contain 1% penicillin/streptomycin, and cells were cultured at 37 °C in a 5% CO_2_ atmosphere. Captopril, lisinopril, olmesartan, and telmisartan were purchased from Biomol (Hamburg, Germany). Drugs were dissolved in 100% DMSO and further diluted in media (c_stock_ = 25 mM, maximum DMSO concentration during experiments 0.3%).

### 2.2. Determination of mRNA Expression

Caco-2 cells were incubated with increasing concentrations of olmesartan/telmisartan (0, 1, 10, 50 µg/mL), captopril (0, 1, 5, 10 µg/mL), lisinopril (0, 0.005, 0.05, 0.5 µg/mL), or vehicle (DMSO) for 24 h, 48 h, or 72 h. The mRNA was extracted by RNeasy Mini Kit (Qiagen, Hilden, Germany) according to the manufacturer’s instructions. The cDNA synthesis was performed using a First Strand cDNA-Synthesis kit (Thermo Scientific, Schwerte, Germany) including random hexamers. The expression levels of ACE2 and β-actin were determined using SYBR Select Mix (Applied Biosystems, Austin, TX, USA) with QuantStudio™ 12K Real-Time PCR System (Applied Biosystems, Austin, TX, USA). For human ACE2 mRNA detection, the forward primer 5′-AATGGGTCTTCAGTGCTCTC-3′ and reverse primer 5′-GAGCCTCTCATTGTAGTC-3′ and for human β-actin the forward primer 5′-CCAACCGCGAGAAGATGA-3′ and the reverse primer 5′-CCAGAGGCGTACAGGGATAG-3′ were used. Relative mRNA expression was determined using the comparative CT (cycle threshold) method, normalizing relative values to the expression level of human β-actin.

### 2.3. Determination of Protein Expression

Caco-2 cells were incubated with increasing concentrations of olmesartan/telmisartan (0, 1, 10, 50 µg/mL), captopril (0, 1, 5, 10 µg/mL), lisinopril (0, 0.005, 0.05, 0.5 µg/mL), or vehicle (1% DMSO) for 24 h, 48 h, or 72 h. Cells were harvested, cell pellets were homogenised and sonicated in RIPA-buffer (25 mM Tris-HCl (pH 7.6)), 1% sodium deoxycholate, 0.1% SDS, 1% IPEGAL, 150 mM NaCl, Roche cOmplete™ Mini tablets (Sigma Aldrich, Schnelldorf, Germany). The homogenate was centrifuged; the supernatants were collected and stored at −20 °C. Protein concentrations were assessed using the bicinchoninic acid assay (Thermo Fisher Scientific, Schwerte, Germany). Fifty micrograms of total protein extract were separated electrophoretically by 10% SDS-PAGE and electroblotted onto nitrocellulose membranes (Amersham Life Science, Freiburg, Germany). Membranes were blocked in 5% non-fat dry milk in TBS supplemented with 0.05% Tween 20 and incubated with the respective primary antibody directed against ACE2 (1:1000) and ß-Actin (1:10,000). All antibodies were diluted in 1% BSA in 0.1% Tween 20 in TBS. Membranes were washed three times with 0.05% Tween 20 in TBS and then incubated with an anti-rabbit AF488 or anti-mouse AF546 antibody in 1% BSA in 0.1% Tween 20 in TBS. Membranes were washed three times with 0.05% Tween 20 in TBS and were analysed on ChemiDoc™ MP Imaging System from Bio-Rad Laboratories (Hercules, CA, USA). The mouse monoclonal anti-β-actin antibody was purchased from Sigma Aldrich (Schnelldorf, Germany), the rabbit polyclonal anti-ACE2 antibody from Abcam (Berlin, Germany), and the anti-rabbit AF488 and anti-mouse AF546 antibodies from Thermo Scientific (Schwerte, Germany).

### 2.4. Infection Assay

Caco-2 cells were seeded in clear 96-well plates (F-bottom) and incubated until they reached full confluence. The cells were subsequently treated with serial dilutions of the investigated compounds or remdesivir as a positive control (in triplicates per concentration) for 72 h before infection in MEM medium containing 10% FCS. Following this pre-incubation, the cells were treated with the same compound dilutions in MEM medium containing only 1% FCS, to prevent the inhibition of infection by components of FCS [12]. Moreover, soluble ACE2 can effectively block the binding of SARS-CoV S-protein to membrane-bond ACE2 and thereby prevents SARS-CoV entry [13,14], which is possibly transferable to SARS-CoV-2. Since FCS contains soluble ACE2, a reduced FCS concentration possibly intensifies the infection of Caco-2 cells [15]. The cells were infected with SARS-CoV-2 at a multiplicity of infection (MOI) of 0.01 for 24 h in the presence of the compounds. After the 24 h incubation, the medium was removed and the cells fixed with acetone/methanol mixture (40:60) for 10 min before blocking with blocking solution (2% BSA, 5% goat serum, 0.01% thimerosal) over night at 4 °C.

### 2.5. Spike Protein Immunostaining

Fixed and blocked cells were incubated with an anti-SARS-CoV-2 spike antibody (rabbit, 1:1500, SinoBiological (Eschborn, Germany)) for 1 h at 37 °C, washed twice, and subsequently incubated with an HRP-coupled anti-rabbit antibody (goat, 1:1000, Jackson Immunoresearch (Cambridgeshire, UK)) for 1 h at 37 °C. After another two washing steps, the cells were stained by the addition of 3-amino-9-ethylcarbazole (AEC) solution for 10 min, washed and the percentage of spike positive area was detected in the BIOREADER-7000 F-z device. The percentage of spike positive area per well was quantified and the values of the compound-treated samples were normalized to the virus control without compounds (=100%). Values lower than 100% represent virus inhibition, while values above 100% could indicate virus promotion.

### 2.6. Cell Viability Assay

To determine the viability of human Caco-2 cells, an MTT assay was performed in parallel at the same compound dilutions as the infection assay but without the addition of SARS-CoV-2. The infection assay and the cytotoxicity assay were stopped at the same time and 25 µL of MTT solution was added to the cytotoxicity plates and incubated at 37 °C for 4 h. A measure of 100 µL of acidified sodium dodecyl sulfate (SDS) (870 mM, pH 4.7 in water:DMF (50:50)) was added and plates were incubated overnight at 37 °C. Absorbance at 560 nm with a reference wavelength of 620 nm was measured using a TECAN GENios Basic. To calculate the cell viability, all absorbance values were blank subtracted, the values of untreated cells were set to 100% and the compound-treated samples were correlated to them.

### 2.7. Cell Barrier Assay

To generate the Caco-2 cell barrier, 100,000 Caco-2 cells were seeded on ThinCerts^TM^ for 18 days. The cell barriers were pretreated with olmesartan, telmisartan, captopril (each 10 µg/mL), lisinopril (0.5 µg/mL), remdesivir (positive control; 0.6 µg/mL), or vehicle (DMSO) for 72 h. The cells were infected with SARS-CoV-2 virus (MOI 0.1) or were not infected in the presence of the drugs or vehicle (DMSO) and incubated for 24 h, 48 h, and 72 h. The cell barrier integrity was evaluated by the detection of the transepithelial electrical resistance (TEER) with Millicell-ERS (Electrical Resistance System, Millipore) before the pretreatment (−72 h), at the beginning (0 h) and after (24 h, 48 h, 72 h) virus infection. The TEER values at time points 24 h, 48 h, and 72 h were related to the values at time point 0 h.

### 2.8. Statistics

Results are presented as means ± standard errors (SEM). The data was analysed with two-way ANOVA and with Dunnett’s multiple comparisons test. For all calculations and for the creation of graphs, GraphPad Prism 8 was used and *p* < 0.05 was considered the threshold for significance.

## 3. Results

### 3.1. ACE2 mRNA and Protein Expression Is Not Regulated by ACE Inhibitors and AT_1_ Antagonists

**Figure 1:**
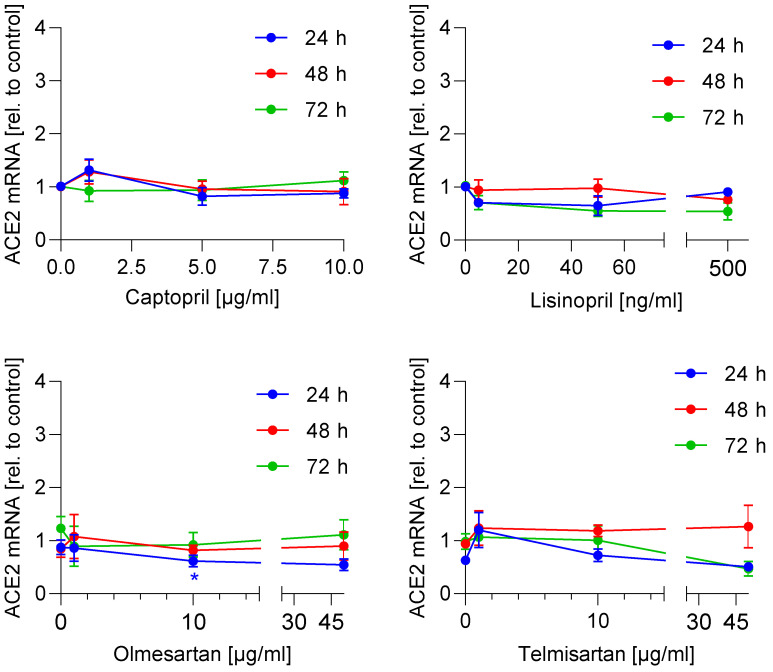
**Effect of ACE-inhibitors and AT_1_R antagonists on ACE2 mRNA expression.** Caco-2 cells were incubated with ACE-inhibitors, AT_1_R antagonists, or vehicle in the indicated concentrations and time points. The mRNA expression was determined using quantitative PCR, normalized to β-actin and drug-treated samples were related to the vehicle treated samples (control) to obtain the fold induction. The experiment was achieved in three biological and three technical replicates. The means of three technical replicates are shown and used for statistical analysis (two-way ANOVA with Dunnett’s multiple comparisons test). * *p* < 0.01 show statistically significant difference between drug-treated and vehicle-treated samples.

**Figure 2:**
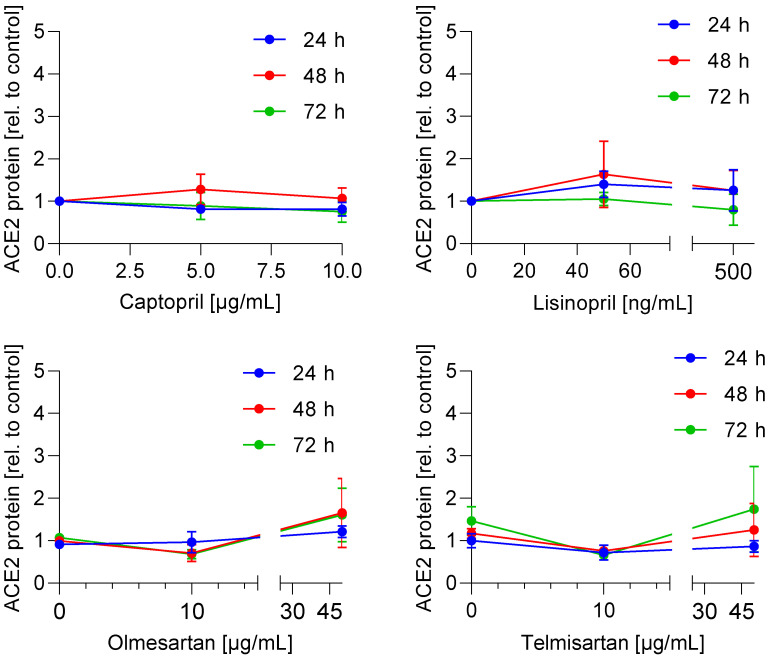
**Effect of ACE-inhibitors and AT_1_R antagonists on ACE2 protein expression.** Caco-2 cells were incubated with ACE-inhibitors, AT_1_R antagonists, or vehicle in the indicated concentrations and time points. The protein expression was determined using Western blot technology, normalized to β-actin and drug-treated samples were related to non-treated samples to obtain the fold induction. The experiment was achieved in three biological replicates. Two-way ANOVA with multiple comparisons test was used to analyse statistical difference between drug-treated and vehicle-treated samples.

It is well described that SARS-CoV-2 uses ACE2 as an entry receptor [16], leading to proposals that ACE inhibitors and/or AT_1_R antagonists may modulate the entry of SARS-CoV-2 virus via increased expression of ACE2. Therefore, we investigated whether ACE inhibitors (captopril, lisinopril) or AT_1_R antagonists (telmisartan, olmesartan) regulate ACE2 mRNA expression. For that purpose, Caco-2 cells were incubated with increasing drug concentrations for 24 h, 48 h, and 72 h. The selected concentrations are in the range of c_max_ plasma levels in humans [17,18,19,20,21,22], high enough to inhibit ACE [23] or block AT_1_R receptor [24] and typically used for in vitro experiments [25,26,27]. However, there was no concentration-dependent alteration of the ACE2 mRNA expression (Figure 1). A measure 10 µg/mL telmisartan reduced the ACE2 mRNA expression slightly after 24 h. Next, we investigated whether this translates into a reduced protein expression level. Caco-2 cells were incubated with increasing concentrations of the drugs for 24 h, 48 h, and 72 h. No significant effect of AT_1_R antagonists and ACE inhibitors on the ACE2 protein expression in Caco-2 cells was observed (Figure 2 and Figure S1). These data indicate that the ACE inhibitors and AT_1_ antagonists do not modulate the efficiency of SARS-CoV-2 entry into Caco-2 cells by means of inducing upregulation in ACE2 levels.

### 3.2. ACE Inhibitors and AT_1_ Antagonists Have No Effect on Caco-2 Cell Viability

**Figure 3:**
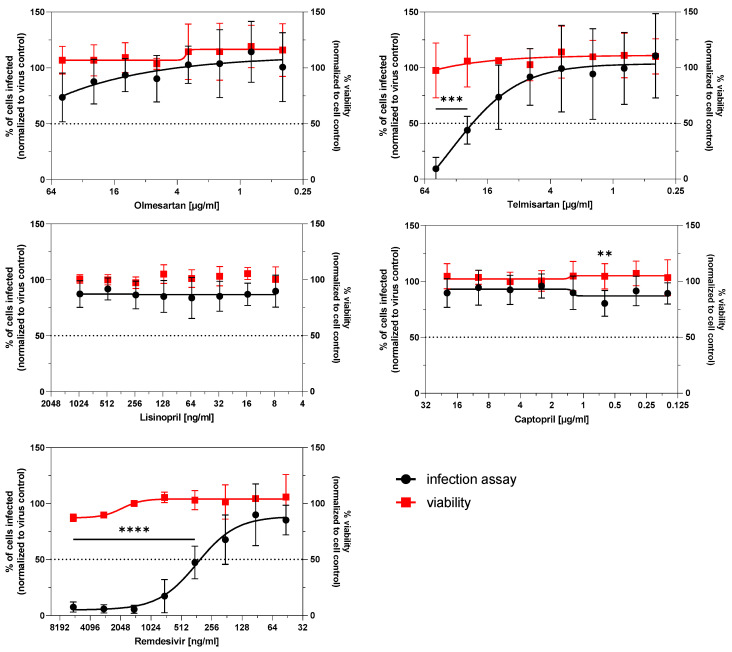
Effect of ACE-inhibitors and AT_1_R antagonists on Caco-2 viability and SARS-CoV-2 virus replication. Fully confluent Caco-2 cells were incubated with ACE-inhibitors, AT_1_R antagonists or vehicle (DMSO) in the indicated concentrations for 72 h and subsequently infected with SARS-CoV-2 at an MOI of 0.01 for 24 h. Afterwards, the cells were fixed and stained via immunohistochemistry against SARS-CoV-2 spike protein. The percentage of spike-positive area per well was quantified and the values of the compound-treated samples were normalized to the virus control without compounds (black circles). For cell viability experiments (red squares), the cells were treated as mentioned above but not infected. After 96 h, the cell viability was determined via MTT assay. The experiments were achieved in three biological replicates. For statistical analysis, one-way ANOVA with multiple comparisons test was used. ** *p* < 0.01, *** *p* < 0.001, **** *p* < 0.0001 show statistically significant differences between drug-treated and vehicle-treated samples.

To determine any underlying effects of ACE inhibitors and AT_1_R antagonists on cell viability, Caco-2 cells were treated for 72 h with increasing concentrations of the drugs, and the viability was assessed by MTT. Interestingly, the drugs did not influence Caco-2 viability (Figure 3).

### 3.3. Infection Potential of SARS-CoV-2 Is Not Affected by ACE Inhibitors and AT_1_R Antagonists

To investigate whether the ACE inhibitors and AT_1_R antagonists inhibit or foster the virus infection, Caco-2 cells were preincubated for 72 h with the test drugs and infected with SARS-CoV-2 with an MOI of 0.01 for 24 h. The infection level of Caco-2 cells by SARS-CoV-2 were determined by staining of the spike protein of SARS-CoV-2. The antiviral compound remdesivir inhibited the virus replication with an IC_50_ of around 0.36 µg/mL (≈650 nM), in accordance with other published studies [28,29]. Olmesartan, lisinopril, and captopril did not influence the virus infection. Interestingly, 25 µM telmisartan significantly reduced the virus replication and 50 µM telmisartan almost completely inhibited the infection of Caco-2 cells (Figure 3).

### 3.4. SARS-CoV-2-Induced Disintegration of Caco-2 Cell Barrier Is Not Prevented by ACE Inhibitors and AT_1_R Antagonists

**Figure 4:**
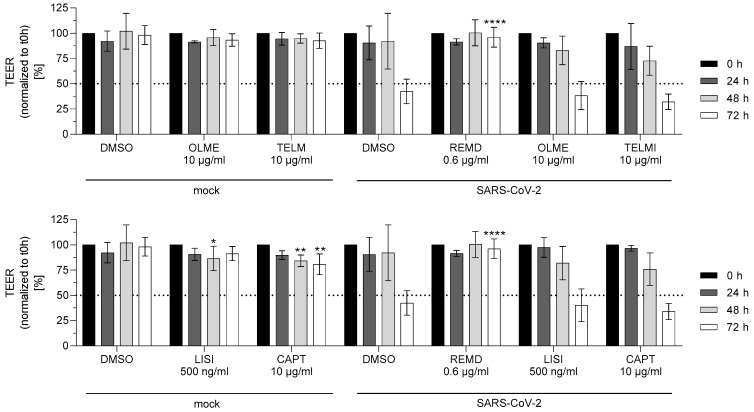
Effect of ACE-inhibitors and AT_1_r antagonists on Caco-2 cell barrier integrity. Caco-2 cell barrier was preincubated at day 18 with ACE-inhibitors, AT_1_R antagonists, or vehicle (DMSO) in the indicated concentrations for 72 h, infected with SARS-CoV-2 (MOI 0.1) and incubated for the indicated time points. The cell barrier integrity was determined by measurement of the TEER value. The TEER values obtained after 24 h, 48 h, and 72 h were normalized to the TEER value obtained at 0 h. The experiment was carried out in three biological replicates. For statistical analysis, one-way ANOVA with multiple comparisons test was used. * *p* < 0.01, ** *p* < 0.01, **** *p* < 0.0001 show statistically significant differences between drug-treated and vehicle-treated samples at the same time point.

To determine whether the ACE inhibitors and AT_1_R antagonists have a protective effect for the cell barrier integrity, the Caco-2 cell barrier model was used. A Caco-2 cell barrier system, generated over 18 days, was exposed with the test drugs for 72 h and subsequently infected with SARS-CoV-2 at a MOI of 0.1. The cell barrier integrity was determined at various time points by measuring the TEER. The degree of infection was evaluated after the experiment via immunohistochemically SARS-CoV-2 spike protein staining (Figure S2). SARS-CoV-2 infection led to a time-dependent reduction of the cell barrier integrity shown by a decreasing TEER value. The virus replication-inhibiting positive control remdesivir (0.6 µg/mL) prevented the reduction of the TEER values. However, none of the ACE inhibitors and AT_1_R antagonists prevented the SARS-CoV-2-induced reduction of the TEER values (Figure 4).

## 4. Discussion

Within this study, it was demonstrated that ACE2 mRNA and protein expression is not modulated by ACE inhibitors and AT_1_R antagonists in Caco-2 cells. In addition, these drugs do not show an induction of the SARS-CoV-2 level of infection in Caco-2 cells. Interestingly, telmisartan even reduced the SARS-CoV-2 level of infection at high concentrations. Moreover, SARS-CoV-2 infection disturbed Caco-2 cell barrier integrity, which was not counteracted by the ACE inhibitors or AT_1_R antagonists. The data indicate that modulators of the angiotensin renin system do not have either positive or negative effects on SARS-CoV-2 infection and SARS-CoV-2-induced disturbance of cell barrier integrity in pharmacologically relevant dosages.

It was hypothesized that modulators of the angiotensin–renin system increase the infection with SARS-CoV-2 via increased ACE2 expression, since SARS-CoV-2 entry into cells depends on ACE2 [9]. However, our results revealed that ACE inhibitors and AT_1_R antagonists have no effect on the ACE2 mRNA and protein expression in the colon epithelial cell line Caco-2 and the infection rate was also not influenced by these drugs (except by telmisartan). In contrast to our in vitro results using a human cell line, in vivo results from other research groups showed that modulators of the angiotensin–renin system regulate ACE2 expression. Lisinopril administered to rats over a period of 12 days led to a three- and fivefold induction, respectively, of ACE2 mRNA expression in heart tissue [30]. In a rat animal model of myocardial infarction, treatment with olmesartan for 28 days induced a three- or 2.5-fold respective increase in ACE2 mRNA expression in heart tissue, whereby the induction of the myocardial infarction alone had no effect on ACE2 mRNA expression [31]. A 3-week treatment with captopril in rats showed a modest 1.4-fold increase in ACE2 mRNA expression in lung tissue at very high concentrations (40 mg/Kg) [32]. The different results of the in vivo models and our in vitro model may be explained by the difference of the exposure times to the compounds. A maximum of 72 h was used in our in vitro model, whereas the rats were treated for a period of 12–28 days. In addition, the increased mRNA expression in the animal model also could lead to an increased protein expression. The protein expression was not investigated in these studies, but the ACE2 activity (ability to cleave a synthetic 7-Methoxycoumarin-4-acetic acid-based peptide [32] or to convert angiotensin 2 to angiotensin 1–7 [30]). Captopril induces ACE2 activity, which may be due to increased ACE2 protein expression [30,32]. Lisinopril does not have an effect on ACE2 activity [30]. Another potential reason for the different results presented here and in the published in vivo studies could be attributed to the different tissue types that were analyzed. Two of the in vivo studies focused on the heart tissue with the aim to analyze the effects of antihypertensive agents on the heart, whereas in our study, the barrier function of intestinal epithelial cells for SARS-CoV-2 was investigated [11]. Interestingly, the ACE2 mRNA expression is higher in the intestine in contrast to heart or lung tissue [33]. It would therefore be useful to investigate the effect of the modulators of the angiotensin–renin system on lung epithelial cells in follow-up studies. The effect of captopril on ACE2 mRNA expression in lung cells has already been investigated. It has been found that 0.05 µg/mL captopril induces a transient 1.25-fold increase in ACE2 mRNA in a human lung cell line (A549) after 24 h, which was no longer detectable after 48 h [32].

Our results do not support the hypothesis that modulators of the angiotensin–renin system may increase the infection with SARS-CoV-2 via increased ACE2 expression. In contrast, telmisartan reduced the viral infection. Since none of the other AT_1_R antagonists could induce this effect, it can be assumed that this is not a class effect, and the anti-viral effect is not necessarily related to antagonization of the AT_1_R. Similarly, olmesartan showed anti-SARS-CoV-2 activity (IC_50_ = 1.8 μM and 5.8 μM, respectively) in the Vero E6 kidney cell line [34]. However, the studies of Alnajjar et al. were carried out in different cell types and with longer incubation periods. Since telmisartan also activates other targets (e.g., PPARγ [35], Cyp2J2 [36], P-glycoprotein [37]), a modulation of these targets may be responsible for preventing the entry of the virus. It cannot be ruled out that telmisartan has a direct effect on the virus, but further studies are needed to elucidate the mechanism by which telmisartan reduces SARS-CoV-2 infection.

Telmisartan is administered at a dose of 40 mg/day for hypertension and 80 mg/day for cardiovascular prevention. Pharmacokinetic studies over 28 days patients with high blood pressure who received 40 or 80 mg/day telmisartan, reports a c_max_ of 0.46 or 1.0 µg/mL plasma level [19]. This indicates that the plasma concentration in high blood pressure patients would not be sufficient to achieve a substantive anti-viral effect. To investigate the effects of telmisartan in COVID-19 patients, a phase-II study has been carried out (ClinicalTrials.gov Identifier: NCT04355936), the results of which are not yet available [38]. Recent data of COVID-19 patients who received ACE inhibitors or AT_1_ antagonists as an accompanying therapy for their high blood pressure have been reported, with some studies demonstrating a positive effect on the survival rate of COVID-19 patients, and others exhibiting no benefit [39,40,41]. However, there are indications in which ACE inhibitors and AT_1_ antagonists have differential effects and should therefore be evaluated separately from one another [35].

It was suggested that SARS-CoV-2 induces vascular dysfunction via an altered angiotensin II/angiotensin 1–7 ratio. Angiotensin I is cleaved to angiotensin II by ACE, which is further converted to angiotensin 1–7 by ACE2. The major physiological action of angiotensin II including vascular dysfunction, inflammation, fibrosis, and vasoconstriction are mediated via AT_1_Rs [42]. AT_2_R-mediated actions such as vasoprotection oppose those of the AT_1_R. Angiotensin 1–7 mediates via Mas1 receptor and Mas-related-G-protein-coupled receptor (MrgD) vasoprotective effects [43]. An increased level of angiotensin II and a decreased level of angiotensin 1–7 by the upregulation of ACE may induce vascular dysfunction and reduce vasoprotective mechanisms. ACE inhibitors may prevent the imbalance of the angiotensin II/angiotensin 1–7 ratio and thereby counteract resulting vascular dysfunction. Moreover, AT_1_R antagonists can directly prevent vascular dysfunction by antagonism of angiotensin II/AT_1_R signaling. To mimic the effects of SARS-CoV-2 on barrier dysfunction the Caco-2 cell barrier model was used. Our results demonstrate that SARS-CoV-2 infection reduced the integrity of the Caco-2 cell barrier, as shown by reduced TEER. This is in accordance with the findings of Guo et al. (bioRxiv preprint; doi:https://doi.org/10.1101/2020.09.01.277780; 2 September 2020). They demonstrated with a gut-on-chip model that SARS-CoV-2 infection can impair cell barrier integrity. We observed no protective effects of the angiotensin renin modulators regarding the virus-induced disturbance of the cell barrier integrity in our in vitro model. This was also demonstrated for losartan in a blood–brain barrier model [44]. However, in a challenged blood–brain barrier model, losartan was effective to attenuate the cell barrier loss [45]. Moreover, lisinopril (1 µM), but not telmisartan (1 µM) was able to abrogate the disruption of endothelial cell layers induced by pathogenic *L. interrogans* [46]. These data indicate that possibly AT_1_ antagonists and ACE inhibitors are able to stabilize cell barrier integrity, but in a manner dependent on the cell barrier type and/or on the cause for the disturbance.

One limitation of the used Caco-2 cell barrier model is that the effects of ACE inhibitors on immune cells cannot be addressed. Immune cells are also present in the colon or lung cell barrier, and they are known for their potential to release angiotensin-converting enzyme. Wildhaber et al. observed that intestinal intraepithelial lymphocytes release angiotensin-converting enzyme [47]. In a next step, a more complex cell barrier model with the integration of immune cells could be established and used to evaluate these ACE inhibitors. The Caco-2 model is suitable to test ACE/AT_1_R modulating drugs because their targets are expressed by colon epithelial cells. Howell et al. demonstrated ACE expression on Caco-2 cells [48] and the presence of AT_1_R on colon epithelial cells was shown in an indirect manner. AT_1_R is expressed in the intestinal mucosa surface of rats. In a massive small bowel restriction assay in rats it, was demonstrated that AT_1_R mediates apoptosis, which was prevented by the treatment of losartan. Moreover, in the colon, epithelial cell line HT29 losartan significantly attenuated the increase of apoptosis caused by angiotensin II administration, whereas an AT_2_R inhibitor had no remarkable effect [49].

Remdesivir used as a positive control prevents SARS-CoV-2 infection and disturbance of cell barrier integrity in our test system. However, the study from the WHO Solidarity Trial Consortium found that remdesivir has no effect on hospitalized patients with COVID-19, as indicated by the overall mortality, initiation of ventilation, and duration of hospital stay [50] and is associated with many side effects [51]. Nevertheless, remdesivir is virostatic by blocking the RNA translocation. Cryo-EM reconstruction of a remdesivir-stalled RNA-dependent RNA polymerase complex revealed that three or four remdesivir monophosphates are incorporated in the active site [52]. Therefore, remdesivir serves as a suitable positive control due to its strong virus inhibition in cell culture models [53]. Moreover, in vitro experiments revealed that hepatitis C virus drugs that inhibit papain-like proteases increase remdesivir’s antiviral activity as much as 10-fold. These data indicate that remdesivir, when used in conjunction with SARS-CoV-2 papain-like protease inhibitors, could provide potent efficacy and protection from SARS-CoV-2 [54].

## 5. Conclusions

In conclusion, we observed no influence of ACE inhibitors and AT_1_ antagonists on SARS-CoV-2-induced infection and on SARS-CoV-2-mediated disturbance of cell barrier integrity. Our results indicate that these classes of drugs have neither beneficial nor detrimental effects on COVID-19 patients also undergoing treatment for hypertension. However, the use of this in vitro model has some limitations compared to the clinical setting and therefore without further animal experiments and/or human studies we cannot exclude that modulators of the angiotensin renin system might still affect COVID-19 patients. In addition, no effects on ACE2 expression, SARS-CoV-2-mediated disturbance of cell barrier integrity and SARS-CoV-2 infection rate were detected for ramipril and losartan (Supplemental Figures S1–S4). However, losartan and ramipril are prodrugs that are converted to the active metabolites carboxylic acid and ramiprilate, respectively. For a comprehensive assessment, studies with their active metabolites will also be necessary.

## Data Availability

The raw data supporting the conclusions of this article will be made available by the authors upon reasonable request.

## Supplementary Materials

The following are available online at https://www.mdpi.com/article/10.3390/life11080810/s1, Figure S1: Effect of ACE-inhibitors and AT_1_R antagonists on ACE2 protein expression, Figure S2: Control of infection of Caco-2 cell barriers, Figure S3: Effect of ACE-inhibitors and AT_1_R antagonists on ACE2 mRNA and protein expression, Figure S4: Effects of the prodrugs ramipril and losartan on SARS-CoV-2 replication and Caco-2 cell barrier integrity.
