# Supplementary material for: Characterization of ACE Inhibitors and AT1R Antagonists with Regard to Their Effect on ACE2 Expression and Infection with SARS-CoV-2 Using a Caco-2 Cell Model
Source: Life (Basel). 2021 Aug 10;11(8):810. doi: 10.3390/life11080810 (PMC8399150; doi:10.3390/life11080810)
Supplement: Supplementary file 1 [file life-11-00810-s001.zip › life-1309144-supplementary.pdf]

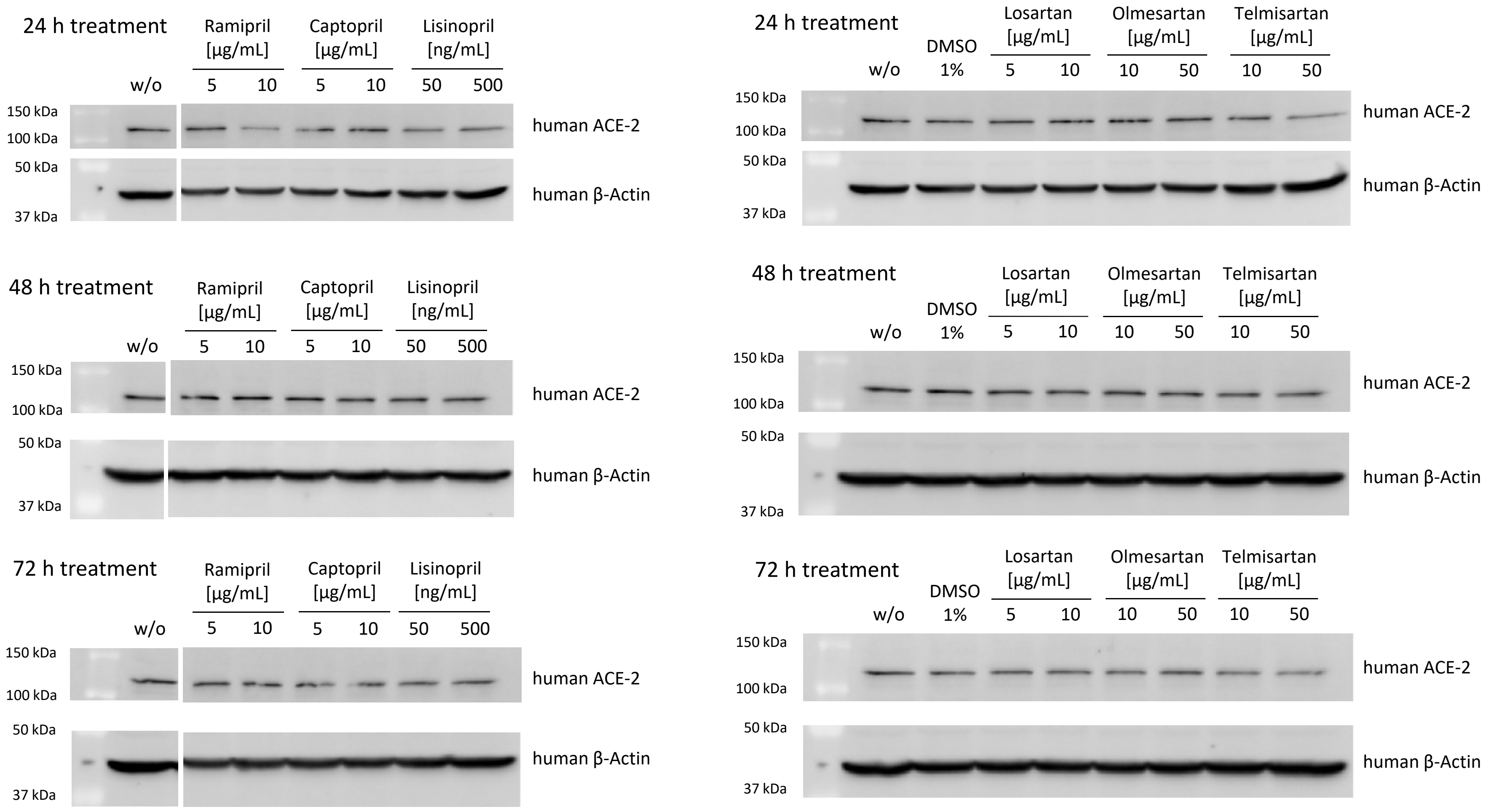

Figure S1: Effect of ACE-inhibitors and AT1 receptor antagonists on ACE2 protein expression. Caco-2 cells were incubated with ACE-inhibitors (ramipril, lisinopril, captopril), AT1 receptor antagonists (telmisartan, olmesartan, losartan) in the indicated concentrations, vehicle (DMSO) or left untreated (w/o) for 24h, 48h or 72h. The protein expression was determined using western blot technology. One representative experiment of three is shown. Molecular weight of human ACE2 ≈ 130 kDa and of human β-actin ≈ 42 kDa. Ramipril was obtained from Sigma Aldrich (Schnelldorf, Germany). Losartan were purchased from Biomol (Hamburg, Germany).

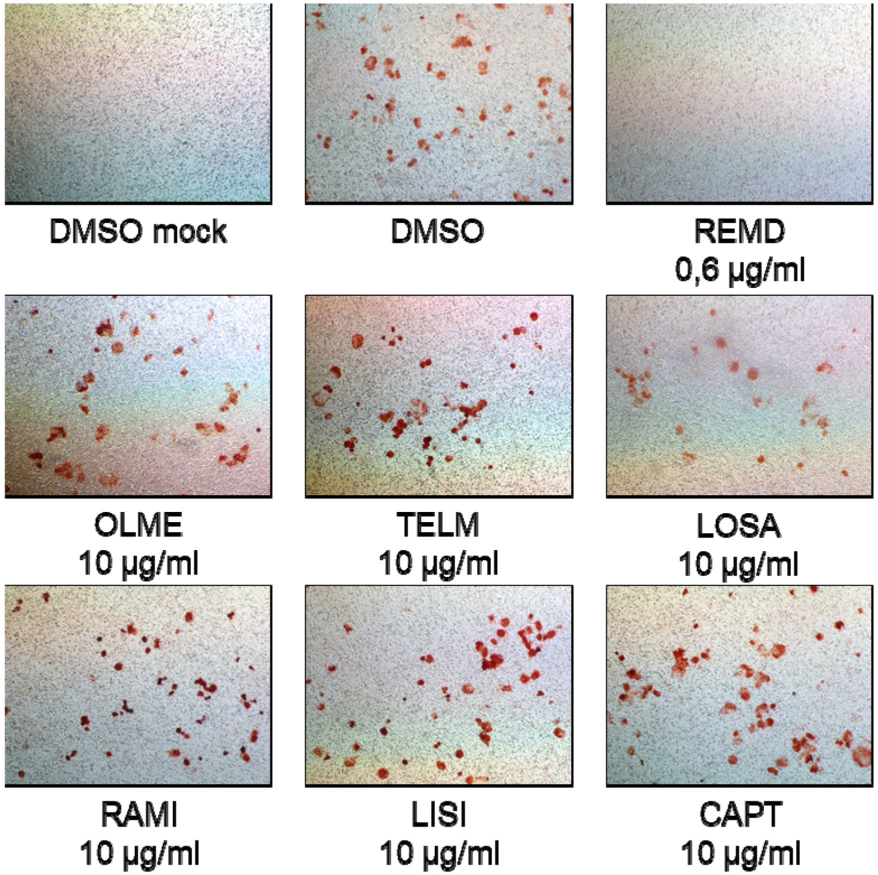

Figure S2: Control of infection of Caco-2 barriers. Caco-2 cell barrier was generated over 18 days and preincubated at day 18 with ACE-inhibitors (ramipril, lisinopril, captopril), AT_1 antagonists (telmisartan, olmesartan, losartan), remdesivir (positive control) or vehicle (DMSO) in the indicated concentrations for 72h. The Caco-2 cell barrier was infected with SARS-CoV-2 (MOI 0.1) or mock infected for 72h. Afterwards, the cells were fixed and stained via immunohistochemistry against SARS-CoV-2 spike protein to visually control the level of infection. Ramipril was obtained from Sigma Aldrich (Schnelldorf, Germany). Losartan were purchased from Biomol (Hamburg, Germany).

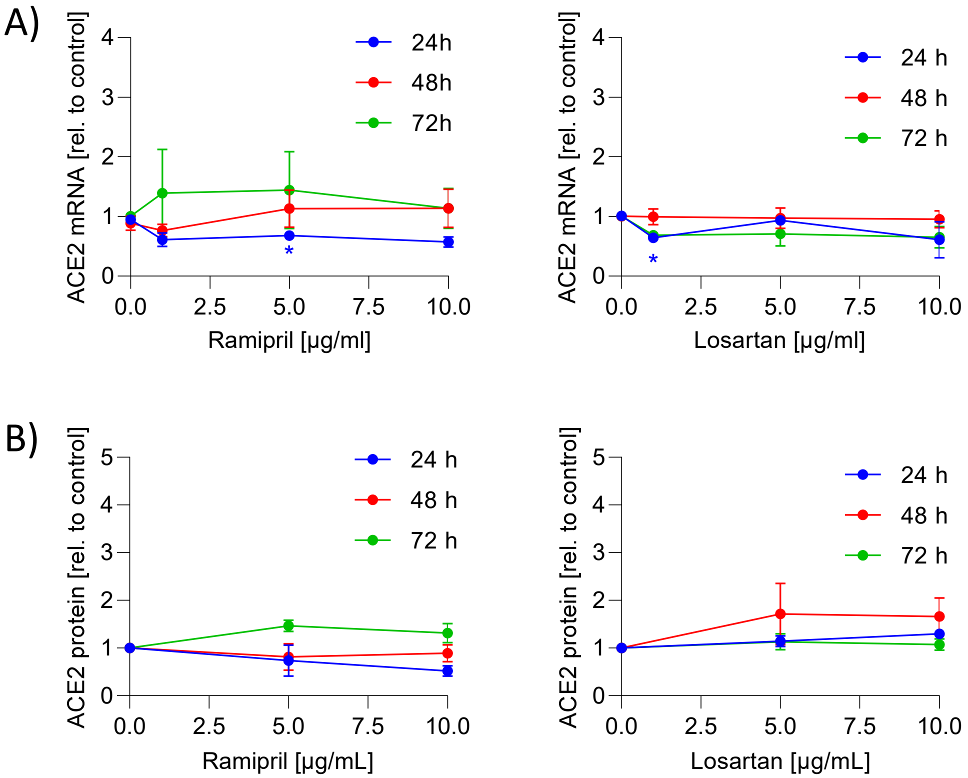

Figure S3: Effect of ACE-inhibitors and AT1 antagonists on ACE2 mRNA (A) and protein (B) expression. Caco-2 cells were incubated with ramipril, losartan or vehicle in the indicated concentrations for 24h, 48h or 72h. The mRNA and protein expression was determined using qPCR or western blot technology. The optical densitometric analysis was achieved with the Image Lab software (Bio-Rad Laboratories, Hercules, USA). The protein and mRNA expression was normalized to β-actin. The protein and mRNA expression of drug treated samples were related to non-treated samples to obtain the fold induction. The experiment was achieved in three biological replicates. Two-way ANOVA with multiple comparisons test was used to analyse statistical difference between drug treated and vehicle treated samples. *$p<0.05$ show statistical significant differences between drug treated and vehicle treated samples. Ramipril was obtained from Sigma Aldrich (Schnelldorf, Germany). Losartan were purchased from Biomol (Hamburg, Germany).

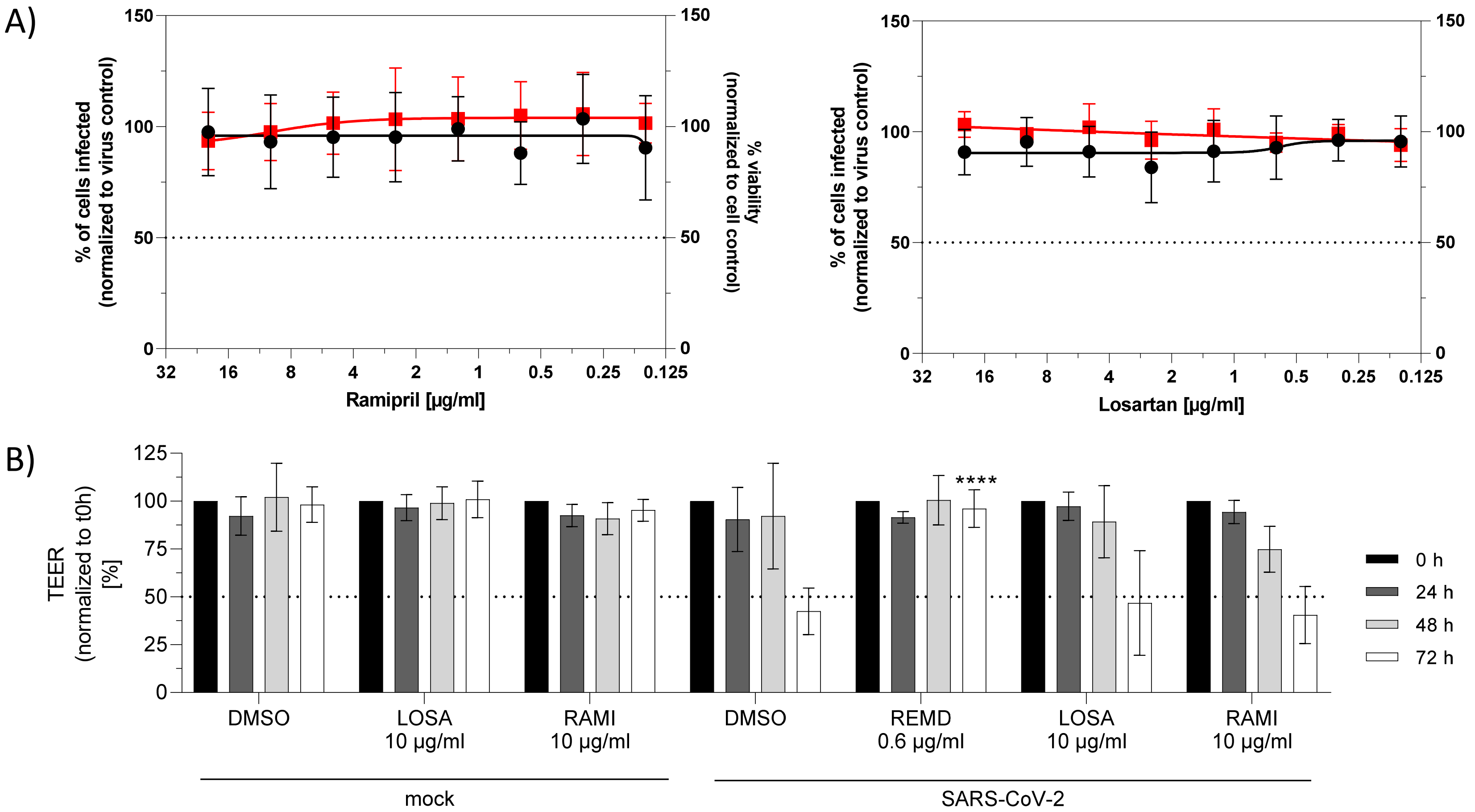

Figure S4: Effects of the prodrugs ramipril and losartan on SARS-CoV-2 replication (A) and Caco-2 cell barrier integrity (B). Caco-2 cells and cell barriers were handled, treated and infected as described for the respective experiments in the materials and methods section. (A) The cells were fixed and stained via immunohistochemistry against SARS-CoV-2 spike protein. The percentage of spike positive area per well was quantified and the values of the compound treated samples were normalized to the virus control without compounds (black circles). For cell viability experiments (red squares), the cells were treated, but not infected. After 96h the cell viability was determined via MTT assay. (B) The cell barrier integrity was determined by measurement of the TEER value. The TEER values obtained after 24h, 48h and 72h were normalized to the TEER value obtained at 0h. The experiments were carried out in three biological replicates. For statistical analysis, one-way ANOVA with multiple comparisons test was used. ****p<0.0001 show statistical significant differences between drug treated and vehicle treated samples. Ramipril was obtained from Sigma Aldrich (Schnelldorf, Germany). Losartan were purchased from Biomol (Hamburg, Germany).
